# Picloram-induced enhanced callus-mediated regeneration, acclimatization, and genetic clonality assessment of gerbera

**DOI:** 10.1186/s43141-021-00269-1

**Published:** 2021-11-15

**Authors:** Saikat Gantait, Manisha Mahanta

**Affiliations:** Crop Research Unit (Genetics and Plant Breeding), Bidhan Chandra Krishi Viswavidyalaya, Mohanpur, Nadia, West Bengal 741252 India

**Keywords:** Auxin, Callogenesis, Indirect organogenesis, Molecular marker, Multiple shoots, Survival

## Abstract

**Background:**

*Gerbera jamesonii* Bolus ex Hooker f. (African daisy) is listed among the top five most important ornamental plants in the global floricultural industry. To satisfy its demand, the floriculture industry relies on reproducible and effective propagation protocol while retaining the genetic uniformity of *G. jamesonii*. The present study, for the first time, reports the potential of picloram for enhanced induction of organogenic calli from leaves of *G. jamesonii* and its high-frequency indirect regeneration.

**Results:**

The fastest induction of calli with maximum fresh and dry weight was recorded in the Murashige and Skoog (MS) semisolid medium supplemented with 1 mg/l picloram. In addition, callus induction was observed in 2,4-dichlorophenoxy acetic acid- and α-napthaleneaceticacid-supplemented media but with delayed response and reduced fresh and dry weight. The proliferated calli were transferred to shoot induction media containing MS salt and 0.5–1 mg/l N^6^-benzylaminopurine, kinetin, or thidiazuron. A mean number of ~6 shoots per callus were developed after 5 days of culture in the MS medium supplemented with 1 mg/l kinetin, with a mean length of 5.2 cm. Successful rooting of shoots was achieved in the MS medium fortified with 1.5 mg/l indole-3-acetic acid, wherein the earliest root initiation (~5 days), as well as the maximum number (~9) and length (~4.8 cm) of roots, were recorded. Complete plantlets were primarily acclimatized in sand before being transferred to a mixed substrate (of soil, sand, tea leaf waste, and cow urine) that secured >90% survival and further growth of the plantlets. Eventually, clonal fidelity of the in vitro regenerants assessed via inter-simple sequence repeats (ISSR) primers exhibited a monomorphic banding patterns that suggested genetic integrity within the plantlets as well as with their mother plant.

**Conclusions:**

The results of the present study should be of interest for commercial propagation and mutagenesis- as well as genetic transformation-related research.

## Background

*Gerbera jamesonii* Bolus ex Hooker f. is commonly known as African daisy that belongs to the family Asteraceae (2n = 2x = 50). It is listed among the top five most important ornamental plants in the global floricultural industry, and the global market demand for gerbera has been ever-increasing over the course of years. It is grown as a cut or pot flower or garden plant under varying climatic conditions. It is native to South Africa, Madagascar, and Indonesia [1]. It is highly valued because of its attractive flowers and thus it is grown for its esthetic value. The flowers with ray and disk florets are available in various colors such as scarlet, yellow, pink, magenta, red, orange, white, etc., and multiple other color patterns. The color of the center (flower eye or the disk floret) can be light or dark. Dark color capitulum provides a contrast against the ray and disk florets and is more attractive that way. In the cut flower market, the vase life of a flower is considered as an important trait for consumers. Most of the commercial cultivars of gerbera have a vase life of 2 weeks or more. Thinner stalk and stem bending are the main factors for lesser vase life [2]. Therefore, improving of quality traits like flower color, size, and longevity is of the need of the hour [3]. Moreover, it has also been considered as a model plant for fundamental research on floral development and secondary metabolism [4].

Conventionally, gerbera is propagated through seeds or vegetatively by division of clumps. Propagation through seeds resulted in undesirable variations in the offsprings since gerbera is cross-pollinated and distinctly heterozygous in nature [5]. Hence, propagation via seed is not commonly used in commercial purpose, while propagation via clump-division is facing drawbacks because of slower multiplication rates and high susceptibility to soil-borne diseases [6]. Therefore, to meet the demand for plantlets and flowers in a commercial scale, a rapid clonal propagation (i.e., in vitro clonal propagation) method is indispensable. With the application of the micropropagation technique, a substantial number of true-to-type plants of a species are obtained within a short span of time under a regulated and disinfected environment [7].

To satisfy the increasing market demand and supply of ready-to-use products, the floriculture industry today relies on such a regeneration protocol that is comparably more reproducible, faster and produces plants with genetic uniformity. Nonetheless, reports on callus-mediated regeneration protocol that guides from the culture establishment to the acclimatization of in vitro regenerated plantlets in gerbera are relatively fewer in comparison to that of the direct regeneration. In general, the indirect regeneration of plantlets via callus is quite crucial for gerbera breeding and has successfully been used to develop protocols for mass propagation, genetic transformation, and induced mutagenesis [8–13]. Different plant parts such as capitulum, flower stalk, and petals were used for indirect regeneration via callus formation in gerbera but the leaf segment proved to be the best [8–11]. Out of the available reports on callus culture and subsequent regeneration in gerbera, none has assessed the potential of picloram as a callus inducer. Most of the reports suggested the use of 2,4-dichlorophenoxy acetic acid (2,4-D), α-napthaleneaceticacid (NAA), N^6^-benzylaminopurine (BAP), etc., in variable combinations and concentrations (i.e., using BAP-NAA in different ratios, like 1:10, 1:1, or 4:1, and using 2,4-D or BAP individually) [8–11], whereas picloram, yet to be tested in gerbera, has proven to be quite effective during induction of organogenic calli in multiple other plant species [14–16]. Considering the potential of picloram and utility of a complete (lab-to-land) protocol, the present study aims to optimize a protocol for enhanced induction of callus and its subsequent regeneration into complete plantlets (with shoots-roots), acclimatization of in vitro regenerated plantlets, and assessment of genetic fidelity of those plantlets using inter-simple sequence repeats (ISSR) marker system.

## Methods

### Collection of plant material, surface disinfection, and preparation of explants

Fresh apical leaves (1.5–2 cm) were collected from a 60-day-old plant growing in a polyhouse. The collected explants underwent surface disinfection (under Laminar Air-Flow chamber) through consecutive treatments utilizing 0.05% (w/v) Bavistin®, 10% (v/v) Tween 20® (polysorbate 20), 20% (v/v) sodium hypochlorite, 1% (w/v) cetrimide (alkyltrimethylammonium bromide), 70% (v/v) ethyl alcohol, and 0.1% (w/v) mercuric chloride (all from Merck Life Sc. Pvt. Ltd., India). Exposure duration of the explants to the majority of the sterilant was 5 min each, except ethyl alcohol (for 30 s). Following completion of the disinfection process, the explants were washed with sterile water.

### Culture media and culture conditions

The Murashige and Skoog (MS) semisolid medium [17] was prepared with the addition of ready-MS salt (HiMedia Laboratories Pvt. Ltd., India) fortified with 3% (w/v) sucrose and 0.7% agar, to serve as basal medium. For the induction of callus and subsequent regeneration of multiple shoots and roots, the MS medium was further fortified with different plant growth regulator (PGR) sources (Sisco Research Laboratories Pvt. Ltd., India) at variable concentrations maintaining the medium pH at 5.7. The growth room was set with 25 ± 1°C temperature, 60% relative humidity, 50 μmol/m^2^/s photosynthetic photon flux density (using cool white fluorescent lights), and 16 h photoperiod. However, during callus induction, initially, the inoculated leaf explants were kept under zero irradiance for 72 h, prior to their transfer to the above-mentioned culture condition.

### Callus induction and proliferation

For induction of callus, the leaf explants were inoculated in the MS medium fortified with 0.5, 1, and 1.5 mg/l 2,4-D, picloram, and NAA, individually. The MS medium without any PGR served as “control”. Explants were horizontally scraped with a scalpel before their inoculation in order to enhance the probabilities of callus induction. Observations were recorded on daily basis for days to callus induction, whereas weight (mg) of fresh and dried (under hot-air oven for 36 h at 45°C) calli were recorded after 6 weeks of culture.

### Shoot initiation from callus

For indirect shoot regeneration, fresh organogenic calli (induced in 1 mg/l picloram-supplemented medium), each weighing 500 mg, were inoculated in the MS medium supplemented with 0.5, 1, and 1.5 mg/l BAP, kinetin, or thidiazuron, wherein the MS medium devoid of PGR served as “control”. Observations were recorded on daily basis for days to fresh shoot initiation, whereas the number and length (cm) of shoots, as well as leaf numbers, were recorded after 6 weeks of culture.

### Rooting of shoots

In vitro shoots were individually inoculated in the MS medium fortified with 0.5, 1, and 1.5 mg/l indole-3-acetic acid (IAA) and indole-3-butyric acid (IBA) for initiation and elongation of roots. The MS medium without any PGR served as “control”. Days taken to root initiation, number, and length of roots were recorded after 4 weeks from the day of inoculation.

### Acclimatization of in vitro regenerants

In vitro regenerated complete plantlets with well-developed shoots and roots were shifted for acclimatization in sand for 2 weeks and then to an equi-volume mixture of soil, sand, tea leaf waste, and cow urine for the next 4 weeks before those were eventually planted in the polyhouse. During initial acclimatization, a transparent polythene sheet was used to cover the whole set-up and frequent spraying of water was done to ensure high humidity. During the initial as well as advanced acclimatization phases, the survival rate of the plantlets was documented. Following complete acclimatization, the plantlets were transferred to larger earthen pots (filled with garden soil) for subsequent growth and flowering.

### Clonal fidelity assessment

To assess the genetic fidelity of the in vitro regenerated and acclimatized plantlets, genomic DNA was collected from leaves of randomly selected plantlets with the aid of GSure® Plant Mini (DNA extraction) Kit (GCC Biotech, Kolkata, India). Following the extraction of DNA, a polymerase chain reaction (PCR) was performed using a thermocycler system (Applied Biosystems^®^ by Life Technologies™, Singapore) for amplification of the fragments. Each 25 μl of PCR mixture included 40 ng template DNA, 0.1 μM of primer [5′(AG)_8_T3′, 5′(CA)_8_AG3′, 5′(AC)_8_C3′, 5′(GGA)_5_3′, and 5′A(GGA)_5_3′, individually], 200 μM of dNTP, 2 mM MgCl_2_, 5 μl 1X Taq polymerase assay buffer, and 0.5 μl Taq polymerase, along with sterile water. PCR was commenced with preliminary denaturation at 94°C for 5 min, and then 35 cycles of denaturation at 94°C for 1 min, 1 min at annealing temperature (40–58°C based on melting point), an extension of 1 min at 72°C, and final extension at 72°C for 10 min. Next, PCR products were resolved using 1.5% (w/v) agarose gel electrophoresis alongside a 100-bp ladder. The gels were then visualized under UV light in the Gel-Doc system (Labmet Asia Pvt. Ltd., Chennai, India), and the amplified bands were scored as “1” for the presence and “0” for the absence for each of the PCR products.

### Data analysis

A completely randomized design was adopted for all the experiments, each of which comprised three replications and 20 samples per replication were involved. Collected data were statistically analyzed using SPSS software package (version 17.0, SPSS Inc., Chicago, IL, USA), and a one-way analysis of variance was formulated to assess statistical significance. Successively, mean±standard error data were compared with Tukey’s test at *P* = 0.05.

## Results

### Callus induction and proliferation

**Fig. 1:**
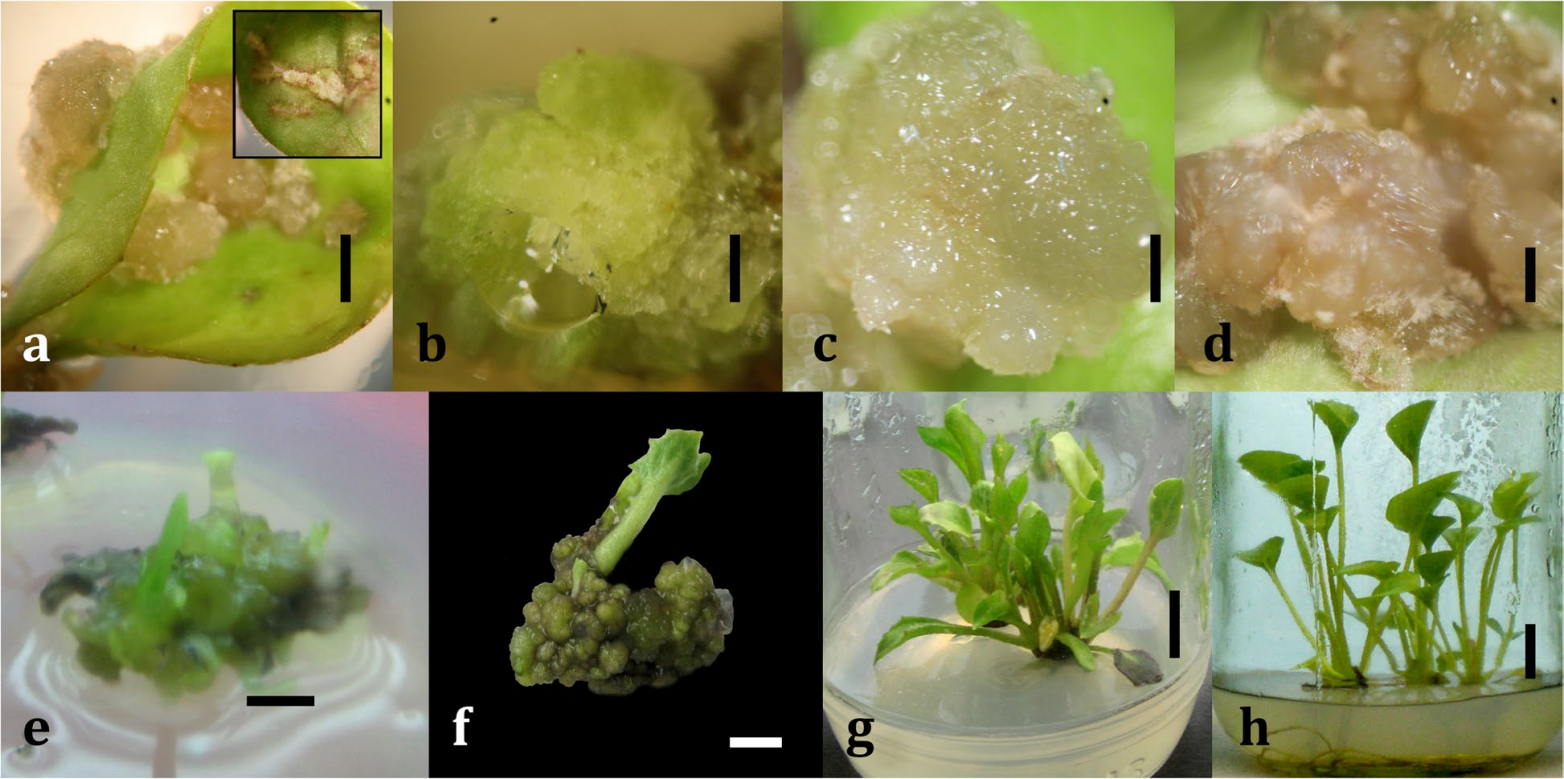
Influence of sources and concentrations of plant growth regulators on callus induction and subsequent indirect plantlet regeneration of gerbera. **a** Induction of callus formation in the form of swelling of the injured sections of leaf due to cellular expansion (*bar* = 5 mm) [*inset:* the injured sections on leaf]; **b** proliferation of organogenic callus (induced from MS + 1 mg/l picloram) (*bar* = 5 mm); **c** proliferation of embryogenic callus (induced from MS + 1 mg/l 2,4-D) (*bar* = 5 mm); **d** proliferation and subsequent necrosis of callus (induced from MS + 1 mg/l NAA) (*bar* = 3 mm); **e** initiation of shoot primordia from nodular callus (induced from MS + 1 mg/l picloram) in MS + 1.5 mg/l kinetin (*bar* = 10 mm); **f** subsequent elongation of regenerated shoots (*bar* = 10 mm); **g** elongation, multiplication, and proliferation of regenerated shoots (*bar* = 25 mm); **h** root initiation and elongation from regenerated and isolated shoots in MS + 1.5 mg/l IAA (*bar* = 25 mm)

The earliest (~8 days) callus induction from the scrapped sites (Fig. 1a) was observed in 1 mg/l picloram-supplemented MS medium, and the slowest callus induction was observed in NAA (Table 1). The “control” medium (without PGR) failed to induce any callus. Out of the three different PGR sources and their nine variable concentrations, MS medium supplemented with 1 mg/l picloram was recorded to be the most effective in terms of callus induction and proliferation. The organogenic callus induced in 1 mg/l picloram recorded a callus diameter of 43.0 ± 2.1 mm with a fresh weight of 651.7 ± 6.9 mg and a dry weight of 66.7 ± 2.3 mg (Fig. 1b; Table 1). In lower (0.5 mg/l) and higher (1.5 mg/l) concentrations of picloram, comparatively less frequent callus induction was observed. On the other hand, 2,4-D performed moderately (Fig. 1c; Table 1) when compared to picloram and performed better when compared to NAA (Fig. 1d; Table 1). NAA at 0.5 mg/l took the longest time (~22 days) to induce callus and resulted in the smallest callus diameter (11.3 ± 0.3 mm) and the lowest fresh weight (211.3±6.8 mg) and dry weight (13.7±0.9 mg), as well as subsequent necrosis of callus. Nonetheless, in the case of both 2,4-D and NAA, it was observed that with an increase in the concentration of these PGRs, a positive effect was noticed in terms of callus proliferation.

**Table 1:** Effect of different sources and concentrations of plant growth regulators (supplemented in the MS semisolid medium) on induction and proliferation of callus from leaf explants of gerbera

PGR (mg/l)	Days to callus induction	Callus diameter (mm)*	Fresh weight (mg)*	Dry weight (mg)*
0.5 2,4-D	12.7±0.7 c	16.0±2.1 e	256.3±6.0 f	21.0±1.2 e
01 2,4-D	12.0±0.6 cd	27.7±1.5 cd	401.7±6.4 d	28.7±1.3 d
1.5 2,4-D	11.3±0.3 cd	33.0±2.6 b	471.3±7.8 c	32.3±1.2 d
0.5 NAA	22.3±1.5a	11.7±1.4 e	211.3±6.8 g	13.7±0.9 f
1 NAA	20.3±0.9 a	16.3±0.8 e	266.3±9.6 f	20.0±1.2 e
1.5 NAA	16.3±0.9 b	23.0±1.7 d	333.7±6.9 e	22.7±1.2 e
0.5 picloram	11.3±0.9 cd	28.3±1.5 bc	452.3±8.5 c	37.3±2.3 c
1 picloram	8.3±0.3 e	43.0±2.1 a	651.7±6.9 a	66.7±2.3 a
1.5 picloram	10.0±0.6 de	32.3±1.4 bc	522.3±4.6 b	45.7±2.1 b
Control	0.0±0.0 f	0.0±0.0 f	0.0±0.0 h	0.0±0.0 g

*Data recorded after 6 weeks of culture

Data (mean±standard error) represent three replicated treatments with 20 leaf explants per replication (control = MS medium without PGR). Data for each *column* followed by the different *letters* are significantly different according to Tukey’s test at *P* = 0.05

### Shoot initiation and proliferation from callus

Shoot regeneration from the callus was studied in different cytokinins such as BAP, kinetin, and thidiazuron at various concentrations. Kinetin outperformed in comparison with BAP and thidiazuron. The earliest shoot initiation (Fig. 1e; Table 2) was observed in 1.5 mg/l kinetin (~5 days) with healthier and maximum number of shoots (5.7±0.3), longest shoots (5.2±0.1 cm), and maximum number of leaves (12.0±1.0) (Fig. 1g; Table 2). It was observed that with an increase in the concentration of kinetin, there was an increase in shoot proliferation and number. Among the tested PGRs, the lowest number of shoots (3.0±0.6) was found in 1 mg/l thidiazuron while the shortest shoot length (2.9 ± 0.2) was seen in 1.5 mg/l BAP. Nonetheless, the “control” medium (without PGR) exhibited the poorest results among all the treatments (Table 2).

**Table 2:** Effect of different sources and concentrations of plant growth regulators (supplemented in the MS semisolid medium) on multiple shoot initiation from organogenic calli of gerbera

PGRs (mg/l)	Days to shoot initiation	No. of shoots*	Shoot length (cm)*	No. of leaves*
0.5 BAP	8.7±0.3 cd	3.7±0.9 b	3.5±0.1 de	7.7±2.1 b
1 BAP	7.0±0.0 de	4.0±0.0 b	3.8±0.2 cd	8.3±0.3 b
1.5 BAP	5.7±0.3 e	4.3±0.3 ab	2.9±0.2 ef	8.7±0.7 b
0.5 kinetin	8.7±0.3 cd	3.7±0.3 b	3.3±0.2 de	8.7±0.3 b
1 kinetin	5.3±0.3 e	4.7±0.7 ab	4.5±0.1 b	9.7±1.2 b
1.5 kinetin	4.7±0.3 e	5.7±0.3 a	5.2±0.1 a	12.0±1.0 a
0.5 thidiazuron	10.3±0.7 c	3.3±0.3 b	3.1±0.1 e	7.3±0.3 b
1 thidiazuron	12.7±0.9 b	3.0±0.6 bc	4.2±0.3 bc	6.7±0.9 b
1.5 thidiazuron	13.0±1.0 b	3.3±0.7 b	3.8±0.3 cd	7.0±1.0 b
Control	25.3±1.8 a	1.7±0.3 c	2.4±0.3 f	3.3±0.7 c

*Data recorded after 6 weeks of culture

Data (mean±standard error) represent three replicated treatments with 20 calli (each weighing 500 mg) per replication (control = MS medium without PGR). Data for each *column* followed by the different *letters* are significantly different according to Tukey’s test at *P* = 0.05

### Root initiation and elongation from shoot

The earliest (4.7 ± 0.7 days) root initiation was recorded in 1.5 mg/l IAA, exhibiting maximum number (8.7±0.9) and length (4.8±0.4 cm) of roots (Fig. 1h; Table 3), while considerably fewer roots (2.0±0.6) were obtained in the “control” medium (without PGR). In the case of IAA, it was observed that with an increase in concentration, there was early root initiation and increase in number and length of roots, whereas in the case of IBA, with an increase in concentration, there was delayed root initiation with fewer and comparatively stunted roots (Table 3). In lesser concentration of IBA (0.5 mg/l), a maximum of around 4 roots with 4.1 cm length were counted from each inoculated shoot.

**Table 3:** Effect of different sources and concentrations of auxins (supplemented in the MS semisolid medium) on root initiation, multiplication, and elongation from shoots of gerbera

PGRs (mg/l)	Days to root initiation	No. of roots*	Root length (cm)*
0.5 IAA	6.3±0.9 de	6.3±0.9 b	3.8±0.2 abc
1 IAA	7.7±0.7 d	8.3±0.9 ab	4.5±0.2 a
1.5 IAA	4.7±0.7 e	8.7±0.9 a	4.8±0.4 a
0.5 IBA	6.3±0.7 de	3.7±0.7 c	4.1±0.2 ab
1 IBA	11.3±0.7 c	2.7±0.7 d	3.2±0.2 bcd
1.5 IBA	14.3±0.7 b	2.3±0.3 e	3.0±0.4 cd
Control	17.0±1.0 a	2.0±0.6 e	2.8±0.3 d

*Data recorded after 4 weeks of culture

Data (mean±standard error) represent three replicated treatments with 20 shoots per replication (control = MS medium without PGR). Data for each *column* followed by the different *letters* are significantly different according to Tukey’s test at *P* = 0.05

### Acclimatization of in vitro regenerants

**Fig. 2:**
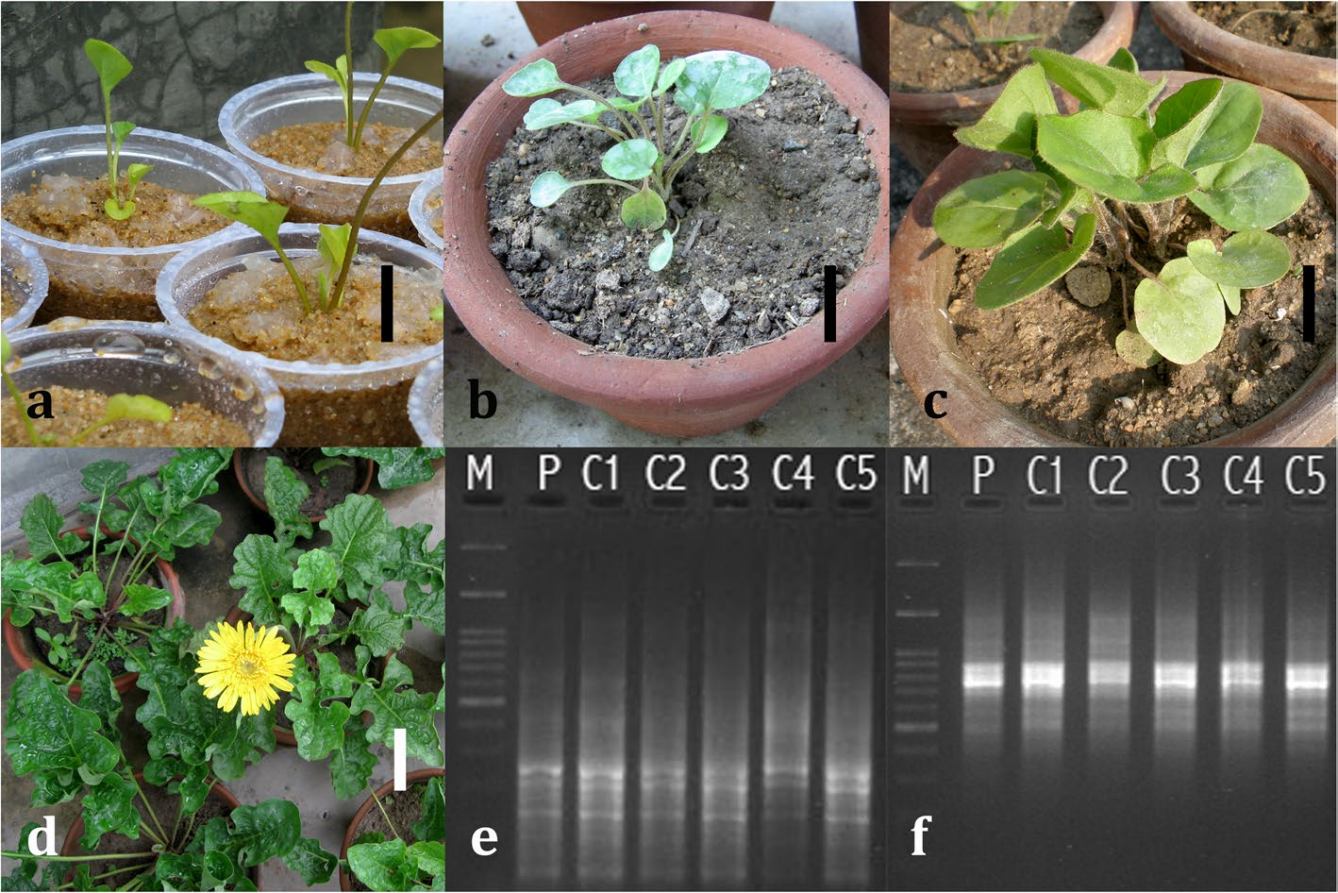
Acclimatization of in vitro regenerants of gerbera. **a** Growth status of plantlets after 1 week of primary acclimatization in autoclaved sand (*bar* = 30 mm); **b** growth status (with the emergence of new leaves and shoots) of plantlets after 2 weeks of acclimatization in a mixture of sand, soil, cow urine, and tea leaf waste (*bar* = 30 mm); **c** growth status (with the emergence and enlargement of new leaves with elongation of shoots) of plantlets after 4 weeks of acclimatization (*bar* = 50 mm); **d** status of vegetative growth, proliferation, and flowering of acclimatized plantlet after 6 months of acclimatization in soil (*bar* = 100 mm); agarose gel electrophoresis of amplified ISSR fragments of regenerants (C1–C5 = regenerants from calli and the mother plant (P) exhibiting monomorphic banding pattern **e** 5′(AG)_8_T3′ and **f** 5′(CA)_8_AG3′ lane M = 100-bp ladder

During acclimatization, >90% survival rate was recorded by the fifth week of the growth period. Sand as a primary substrate proved to be quite effective for the initial phase of acclimatization (Fig. 2a). Later on, during advanced stages of acclimatization (Fig. 2b, c), the blend of soil, sand, tea leaf waste, and cow urine played a crucial role in order to ensure such a high survival rate of the plantlets. After 6 months (of acclimatization) of their (plantlets) transfer to larger earthen pots (filled with garden soil), the plants finally started to bloom (Fig. 2d).

### Clonal fidelity

During PCR and subsequent gel electrophoresis using ISSR primers, all the samples exhibited a monomorphic banding pattern. Each ISSR primer produced distinct bands with different molecular sizes (Fig. 2e, f). The overall banding pattern exhibited by the ISSR primers confirmed that there was no genetic variation within the in vitro regenerated plantlets as well as with their mother plant (from where the explants were collected initially).

## Discussion

The leaf explants of gerbera showed differential callus responses according to the concentrations and combinations of auxins in the culture media. No callus was formed from explants in the MS medium without PGRs. Three different types of callus were observed, i.e., compact-green organogenic (in 0.5–1.5 mg/l picloram-supplemented media), nodular-white embryogenic (in 0.5–1.5 mg/l 2,4-D-supplemented medium), and nodular-brown necrotic (in 0.5–1.5 mg/l NAA-supplemented medium). Different types of callus have been previously induced using different PGRs. For instance, compact-nodular (in 1–4 mg/l BAP-supplemented media), friable-nodular type (in 1–3 mg/l NAA or 2,4-D-supplemented media), and friable type (in 1–3 mg/l IBA-supplemented media) [18]. Nonetheless, in terms of overall callus induction and proliferation, picloram outperformed the other two auxins (in all the aspects of morphological traits, considered in this study). For callus induction in gerbera, a combination of 0.4 mg/l BAP and 4 mg/l NAA was necessary as per earlier report [8], but in another instance, 3 mg/l BAP alone was sufficient to induce callus [9]. In the present study, picloram was used for the first time to induce callus in gerbera and it exhibited significant results when compared with commonly used PGRs such as NAA and 2,4-D. Applications of picloram, either individually or in combination with other PGRs, for callus induction were reported in different plant species, and similar results were found in support of the present experiment. For instance, maximum callus formation was found at 1 mg/l picloram in combination with 5 mg/l BAP in *Simmondsia chinensis* [19]. Likewise, 1 mg/l picloram outperformed NAA and 2,4-D resulting in maximum frequency (90%) of callus induction from pseudostem of *Costus speciosus* [15]. In *Azadirachta indica*, it was found that 1 mg/l picloram with 2 mg/l kinetin resulted in the maximum callus induction with the highest fresh cell weight and dry cell weight [16].

The induced callus showed better shoot proliferation when cultured on a kinetin-supplemented media, followed by BAP- and thidiazuron-supplemented media. Previous studies have shown that 1 mg/l kinetin was more effective than BAP and thidiazuron in enhancing shoot proliferation for *Cucumis sativus* [20] and *Bacopa monnieri* [21].

MS medium supplemented with IAA induced a higher number of roots in a shorter period than IBA. An analogous result was reported in gerbera wherein the maximum number of roots (~5) with a root length of 6.2 cm in a much higher concentration of IAA (3 mg/l) was recorded when compared to NAA [22]. The promotional effect of IAA was also reported by another researcher in gerbera, wherein ½MS medium supplemented with 1 mg/l IAA resulted in 95% rooting frequency [23].

During acclimatization, the utility of tea leaf extract and cow urine proved to be effective for their root-inducing properties and multiple shoot proliferation properties, respectively [24]. Apart from the substrates used, consistent high humidity also played a crucial role in protecting the plantlets from detrimental withering. Intermittent spraying and polyethelene coverage to retain high moisture content are frequently practiced for several horticultural plant species including gerbera [24,^25^–^27^].

In this study, it was found that the regenerated plantlets showed monomorphism during ISSR-mediated fidelity check. Regeneration of genetically true-to-the type plantlets following callus-mediated approach in gerbera was never been reported to date. The confirmation of molecular marker-assisted genetic clonality from callus-regenerated plants in the present study is supported by the earlier observations in multiple other ornamental plant species such as *Clivia miniata* [28], *Elephantopus scaber* L. [29], and *Rhinacanthus nasutus* [30]. In most of the other cases, somaclonal variation was documented from plantlets that derived from indirect organogenesis [31–33], unlike the present study. Hence, the present study could be commercially viable owing to ascertaining the genetic trueness of the in vitro regenerated plantlets.

## Conclusions

In this present study, we have developed an accelerated protocol via indirect regeneration that eventually assured the generation of multiple plantlets retaining their genetic integrity. Picloram proved to be the most effective among all the tested auxins (in different doses). The efficiency of picloram has been documented for the first time in callus induction of gerbera, wherein picloram outperformed 2,4-D and NAA. Successful regeneration of multiple shoots and subsequent roots were also achieved in a short span of time and complete plantlets were fruitfully acclimatized in order to ensure the utility of the present protocol for its commercial application. It is noteworthy to mention that it took as early as 5.5 months from explant inoculation to complete acclimatization. Eventually, the genetic integrity of the plantlets derived via indirect organogenesis substantiates the effectiveness of this protocol. Based on the comprehensive information derived from this study, the developed protocol will be of immense interest for the commercial propagation system as well as in vitro mutagenesis- and genetic transformation-related research approaches in near future.

## Data Availability

The datasets used and/or analyzed during the current study are available from the corresponding author on reasonable request.

## Abbreviations

2,4-D: 2,4-Dichlorophenoxy acetic acid
BAP: N^6^-benzylaminopurine
IAA: Indole-3-acetic acid
IBA: Indole-3-butyric acid
MS: Murashige and Skoog
NAA: α-Napthaleneaceticacid
PGR: Plant growth regulator
